# The kinase homology and dimerization domains in retinal membrane guanylyl cyclase 1 form the binding interface for the calcium sensor GCAP1

**DOI:** 10.1016/j.jbc.2026.113606

**Published:** 2026-09-25

**Authors:** Igor V. Peshenko, Lyuqi Tan, Jai Mahto, Filip Van Petegem, Robert S. Molday, Alexander M. Dizhoor

**Affiliations:** 1 Pennsylvania College of Optometry, Drexel University, Elkins Park, Pennsylvania, USA; 2 Graduate School in Biomedical Sciences and Professional Studies, College of Medicine, Drexel University, Philadelphia, Pennsylvania, USA; 3 Department of Biochemistry and Molecular Biology, University of British Columbia, Vancouver, Canada; 4 Department of Neurobiology and Anatomy, College of Medicine, Drexel University, Philadelphia, Pennsylvania, USA

**Keywords:** guanylate cyclase (guanylyl cyclase), cyclic GMP (cGMP), photoreceptor, retina, retinal degeneration, calcium binding proteins, RetGC, GC-E, GC-A, GCAP, signal transduction, vision

## Abstract

Retinal membrane guanylyl cyclase (RetGC) regulated by guanylyl cyclase activating proteins (GCAPs) enables photoreceptor light sensitivity by producing cGMP to open cyclic nucleotide gated channels. The quaternary structure of the RetGC:GCAP complex remains unresolved. In this study, we conducted site-directed mutagenesis of RetGC1 based on the RetGC1:GCAP1 complex structure generated using AlphaFold 3 in order to identify the interface for GCAP1 on RetGC1. The structure of RetGC1_2_:GCAP1_2_ tetrameric complex from multiple independent trials of AlphaFold 3 modeling consistently positioned GCAP1 in contact with the kinase homology domain (KHD) and the dimerization domain (DD) of RetGC1. Residues in KHD and DD domains predicted to form direct contacts with GCAP1 were substituted to either disrupt or enhance GCAP1 binding by RetGC1 *in vitro*. Three residues in KHD predicted to be a part of the interface structure were most critical for binding GCAP1 based on biochemical analyzes–Asp^639^, Lys^637^, and Met^641^. Several predicted interface residues on DD domain also affected RetGC1 apparent affinity for GCAP1–Ser^819^, Met^823^, Asp^832^, Glu^833^, and Glu^837^. The biochemical evidence supports the AlphaFold 3 structural model of the RetGC1_2_:GCAP1_2_ tetramer, in which the N-proximal semi-globule of each GCAP1 molecule binds KHD domain on one RetGC1 subunit and DD domains on both RetGC1 subunits of the RetGC1 homodimer, indicating that each GCAP1 molecule can directly regulate the activity of RetGC1 by altering the conformation of the cyclase homodimer.

Retinal guanylyl cyclase (RetGC) plays a critical role in rod and cone physiology by generating the phototransduction second messenger, cGMP (1, 2). The main isozyme of RetGC, RetGC1 (encoded by *GUCY2D* in humans and *Gucy2e* in rodents) (3, 4, 5), also known as GC-E or GUCY2D, produces most of the cGMP utilized in rod and cone phototransduction (6). RetGC activity in photoreceptor outer segments in the dark maintains a fraction of cyclic nucleotide-gated channels (CNGCs) in the open state, thereby partially depolarizing the photoreceptor membrane. After illumination, phosphodiesterase 6 (PDE6) hydrolyzes cGMP, resulting in closure of CNGCs and hyperpolarization of photoreceptors, initiating visual perception (reviewed in (1, 2, 7, 8)).

GCAPs (reviewed in (1, 2)) regulate RetGC in the outer segment by mediating negative Ca^2+^-feedback from the changes in free Ca^2+^ concentrations between light and dark (9). Mg^2+^-liganded GCAPs accelerate RetGC activity after illumination, when the influx of Ca^2+^ through CNGCs stops, and shut-off RetGC in the dark, when Ca^2+^ influx through CNGCs resumes, converting GCAPs to their Ca^2+^-liganded state (10). The GCAP-mediated regulation of RetGC by negative Ca^2+^-feedback contributes to shaping rod and cone photoresponses and adaptation to background light (11, 12, 13, 14). A different RetGC-binding protein located predominantly in the inner segment, retinal degeneration-3 (RD3), uncouples RetGC:GCAP complex to prevent its aberrant activation while directing the cyclase delivery to the outer segment (15, 16, 17, 18). Due to the critical role of RetGC1 regulation in photoreceptor function, numerous mutations in the *GYCY2D* gene cause various forms of congenital blindness–Leber congenital amaurosis, congenital night blindness, and dominant cone-rod dystrophy (1, 19, 20, 21). For the same reason, dominant mutations in the *GUCA1A* gene coding for GCAP1 cause progressive cone and cone-rod dystrophy (1, 19, 20).

Despite its importance for photoreceptor physiology and inherited diseases, the structure of the RetGC1:GCAP1 complex remains undetermined. Biochemical evidence indicates that RetGC1 is a homodimer regulated by two molecules of GCAP1 in a cooperative fashion (22, 23, 24, 25, 26, 27). The structure of GCAP1 and its residues creating the interface for the cyclase have been established (28), but the RetGC homodimer still presents a major challenge for structural studies. Parts of its putative structure are limited to modeling utilizing homology between RetGC1 and adenylyl cyclases (22) or chemical cross-linking of RetGC1 residues (29, 30). Yet, the structure of the RetGC1_2_:GCAP1_2_ tetramer currently remains unresolved. In this study, we attempted to define the RetGC1:GCAP1 complex interface using site-directed mutagenesis guided by artificial intelligence–based structural prediction (31, 32). Biochemical evidence in our study identifies the key residues on RetGC1 that form the interface for GCAP1, consistent with the predicted AlphaFold 3 model of the RetGC1_2_:GCAP1_2_ tetramer and suggests the basic mechanism by which GCAP regulates RetGC activity.

## Results

### AlphaFold 3 prediction of the RetGC1:GCAP1 complex

Amino acid sequences of the full-length human RetGC1, including its leader peptide (removed from the mature protein) and of the human GCAP1 were used as the input for AlphaFold 3 (32) to generate the models. Based on previous biochemical studies, RetGC1 is a homodimer of two RetGC1 subunits (22, 23, 24, 27) binding two GCAP1 molecules (26, 33). Only a dimer of RetGC1 can form the active site of the enzyme capable of binding the GTP substrate (22, 23, 24). Therefore, two copies of RetGC1 and GCAP1 were used in the input for the modeling. Although AlphaFold 3 formally allows for inclusion of Ca^2+^, Mg^2+^, GTP and the myristoyl moiety, we found that adding them in the input did not affect the predicted structures of RetGC1, GCAP1, and the RetGC1_2_:GCAP1_2_ tetramer. Consequently, these ligands were omitted from the modeling.

More than 50 independent AlphaFold modeling trials were performed, and the result predicted RetGC1_2_:GCAP1_2_ complex structures were analyzed using a Schrodinger PyMOL software. In all independent models generated by AlphaFold, the tight RetGC1 homodimer had two GCAP1 molecules symmetrically bound on the opposite sides of the RetGC1 dimer (Figs. 1and S1). The following regions with the AlphaFold Predicted Local Distance Difference Test (pLDDT, reference (32) score <70, indicating low confidence in the local structural prediction, were excluded from the subsequent structural analyses: Asn^501^–Asn^551^ in RetGC1, as well as Met^1^**–**Ser^15^ and downstream from Arg^178^ in GCAP1 (Figs. 1 and S1).

**Figure 1:**
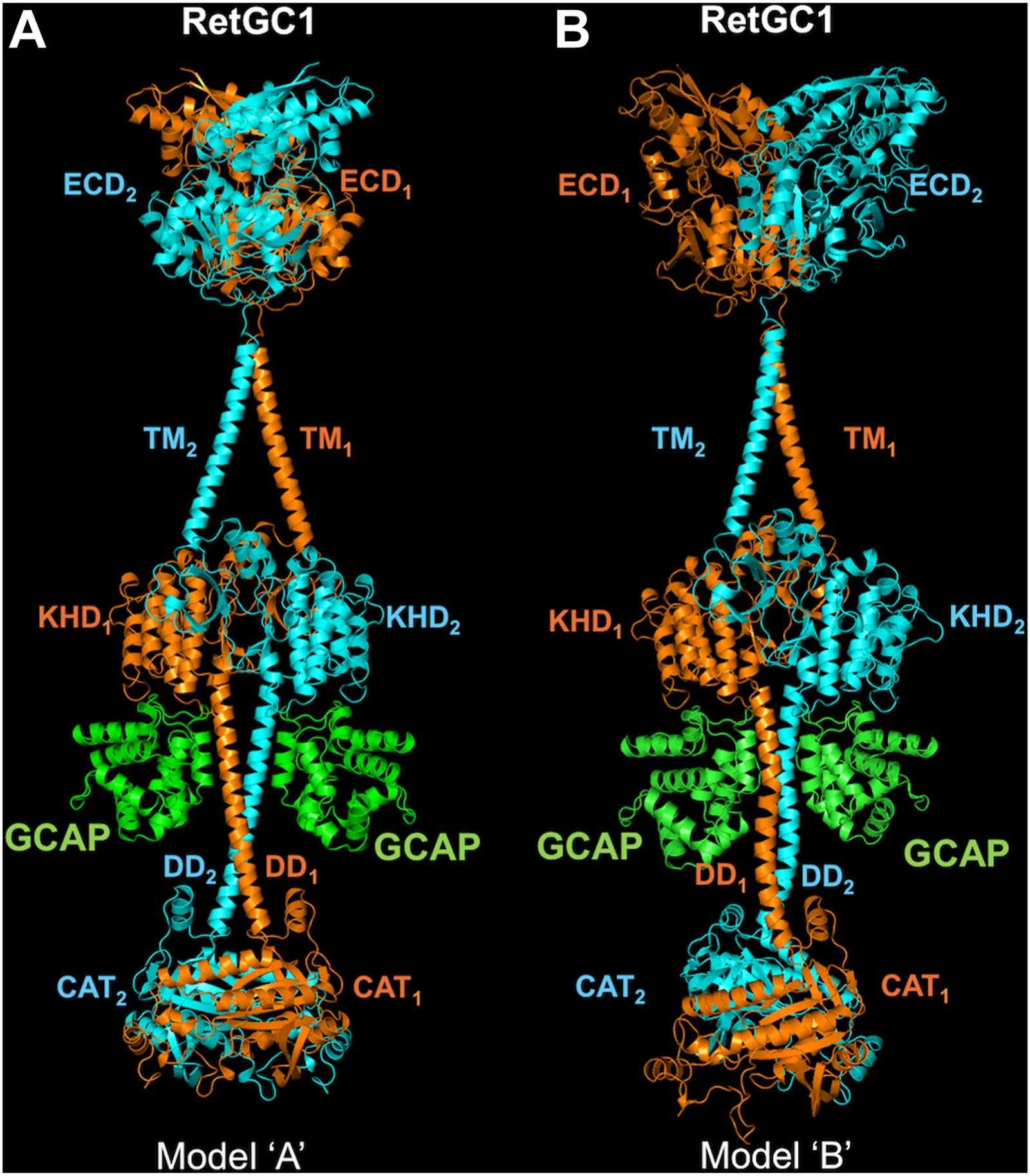
**The structure of RetGC1_2_:GCAP1_2_ tetramer generated by AlphaFold 3.***A*, *B*, representative 3D models, Model A (*left*) and Model B (*right*), each containing two molecules of GCAP1 (*green*) bound to a homodimer of RetGC1 (the two RetGC1 subunits are colored *cyan* and *orange*). The difference between the cytoplasmic portions of the two models is primarily limited to the orientation of the coiled-coils of the DD domains–straight in Model A and kinked/twisted in Model B. See Results for other details. *CAT*, catalytic domain; *DD*, dimerization domain; *ECD*, ‘extracellular’ (intradiscal) domain; *KHD*, kinase homology domain; *TM*, transmembrane domain. Leader peptide (removed from the mature protein), low-confidence structures (pLDDT score <70, ref. (32), and unstructured regions are omitted.

Most of the predicted structures of the RetGC1_2_:GCAP1_2_ tetramer fell into two main visibly distinct groups designated as ‘Model A’ and ‘Model B’ (Figs. 1 and S2), different from each other by the shape of the coiled-coil dimerization domain (DD) of RetGC1. In model A, the coiled-coil DD domain adopted a more straight conformation, similar to previous computational modeling of the RetGC1 coiled-coil DD (23) and the 'canonical' coiled-coil architecture commonly observed in other proteins (34) and in the DD structure of homologous to RetGC1 natriuretic peptide receptor membrane guanylyl cyclase GC-A, recently determined by cryo-electron microscopy (35). By contrast, in Model B, the predicted DD coiled-coil appeared kinked and twisted. Despite the difference in the predicted coiled-coil structures between the two models, the same RetGC1 residues were proximal to GCAP1 in Models A and B (Fig. 2). In both models, each GCAP1 molecule closely interacted with the kinase homology domain (KHD) and DD domains of one RetGC1 subunit, as well as the DD of the second RetGC1 subunit (Fig. S2 and Table 1).

**Figure 2:**
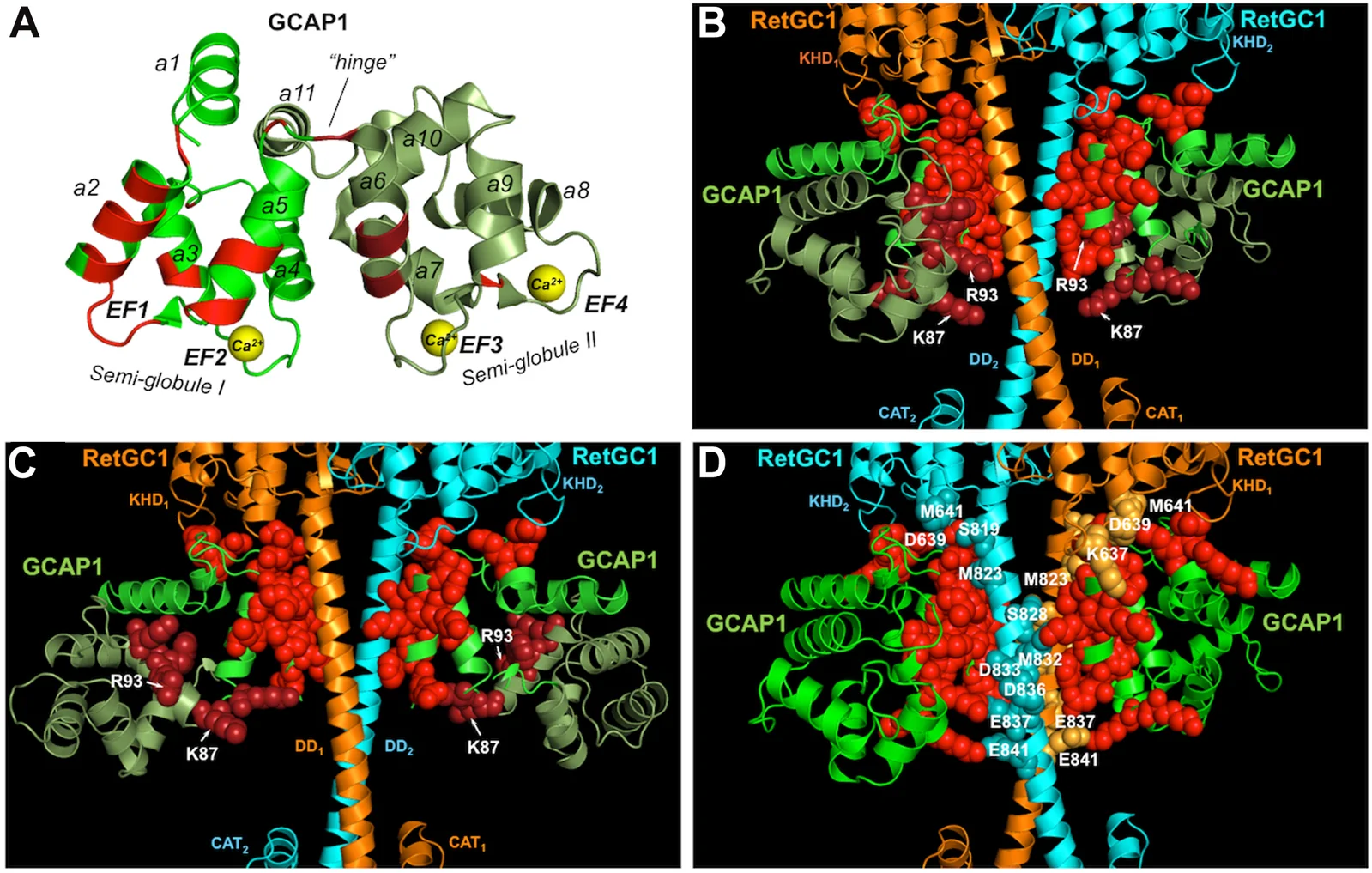
**AlphaFold-predicted RetGC1/GCAP1 binding interface.***A*, three-dimensional structure of GCAP1 based on X-ray crystallography (36): *Myr*, *N*-myristoyl group; *EF1–EF4*, EF-hand domains; *α1–α11*, α-helices numbered sequentially (28) from the amino terminus; *hinge*, the loop between helices α5 and α6 linking the two semi-globular lobes (semi-globule I and semi-globule II). Metal ions in metal-binding EF-hands 2, 3 and 4 are depicted as *yellow sphere*s. RetGC1 binding interface on GCAP1 (28) is marked *red*. *B* and *C*, alternative orientations of the semi-globule II in GCAP1 relative to the RetGC1 DD domain in AlphaFold models; two RetGC1 subunits are shown in *orange* and *cyan*; the semi-globules I and II of GCAP1 are shown in *light green* and *dark green*, respectively; GCAP1 residues identified as part of the RetGC1-binding interface (28) are shown in *spheres*, *bright red* in the semi-globules I or *dark red* in the semi-globules II. *D*, RetGC1 residues predicted to form interface with GCAP1 in the complex and subjected to the mutational analysis are shown as *cyan* and *orange spheres* on the respective RetGC1 subunits.

**Table 1:** Proximal amino acid residues in the AlphaFold-predicted binding interface between RetGC1 and GCAP1

Residue		Distance (mean ± SD), Å		Putative interaction
RetGC1	GCAP1	Model A	Model B	
Lys^637^	Glu^28^	2.51 ± 0.11	3.05 ± 0.49	Salt bridge
Lys^637^	Pro^30^	3.03 ± 0.10	3.53 ± 0.12	Close contact
Lys^637^	Thr^27^	3.08 ± 0.17	2.86 ± 0.27	Close contact
Asp^639^	Ser^31^	2.48 ± 0.04	2.43 ± 0.02	Hydrogen bond
Asp^639^	Pro^30^	3.49 ± 0.09	3.50 ± 0.06	Close contact
Asp^639^	Glu^38^	2.94 ± 0.12	3.15 ± 0.09	Close contact
Asp^639^	Cys^29^	3.35 ± 0.07	3.57 ± 0.07	Close contact
Asp^639^	Gln^33^	3.79 ± 0.15		Close contact
Asp^639^	Glu^28^	3.74 ± 0.09		Close contact
Met^641^	Ser^31^	3.15 ± 0.07	3.36 ± 0.10	Close contact
Met^641^	Tyr^70^	3.28 ± 0.17	Hydrophobic/van der Waals	
Met^641^	Gln^33^	3.66 ± 0.15	2.86 ± 0.33	Close contact
Ser^819^	Ser^31^	2.46 ± 0.25	2.43 ± 0.16	Hydrogen bond
Ser^819^	Gln^33^		3.24 ± 0.15	Close contact
Met^823^	Met^26^	3.54 ± 0.21		Hydrophobic/van der Waals
Met^823^	Pro^30^	3.41 ± 0.22	3.46 ± 0.20	Close contact
Met^823^	Gly^32^	3.29 ± 0.13	3.09 ± 0.05	Close contact
Met^823^	Phe^73^	3.72 ± 0.15		Hydrophobic/van der Waals
Met^823^	Cys^29^		3.76 ± 0.21	Close contact
Glu^832^	Lys^23^	2.55 ± 0.05	2.75 ± 0.42	Salt bridge
Glu^832^	Gln^20^		3.31 ± 0.53	Close contact
Glu^832^	His^19^		3.55 ± 0.20	Close contact
Glu^837^	Gln^90^	2.62 ± 0.22		Hydrogen bond
Glu^837^	Arg^93^	3.36 ± 0.29		Salt bridge
Glu^837^	Lys^85^		2.82 ± 0.25	Close contact

The close (<4Å) atomic distances between RetGC1 and GCAP1 averaged using 16 samples for Model A group and 14 samples for Model B of the RetGC1_2_:GCAP1_2_ tetramer.

Structural alignment of independently generated models showed relatively low variability of the AlphaFold prediction within individual RetGC1 domains. The root-mean-square-deviation (RMSD) calculated for Cα atoms using PyMol in multiple pairwise comparisons of individually aligned domains from Model A (n = 15 comparisons) were 0.27 ± 0.07Å (mean ± SD) for KHD, 0.43 ± 0.18 Å for DD, and 0.63 ± 0.27 Å for CAT domains. The corresponding values for the Model B group (n = 13 comparisons) were 0.21 ± 0.04 Å, 0.47 ± 0.17 Å, and 0.73 ± 0.32 Å, respectively. In contrast to the relatively low structural variability within individual domains, their relative positioning was substantially more variable across independent predictions. When all models were aligned to the KHD domains, the RMSD for the DD and CAT domains increased to 1.60 ± 0.63 and 5.19 ± 2.17 Å, respectively, for Model A and to 1.32 ± 0.51 Å and 5.02 ± 1.81 Å, respectively, for Model B.

The predicted structure of GCAP1 within the RetGC1–GCAP1 complex consisted of two semi-globular lobes connected *via* Gly residue in a “hinge” region between them and closely resembled the structure of GCAP1 established by X-ray crystallography (36) (Fig. 2*A*). RMSD values for the individually aligned first and second semi-globule were 0.73 ± 0.25 Å and 0.70 ± 0.36 Å for Model A (n = 15), and 0.71 ± 0.43 Å and 0.93 ± 0.75 Å for Model B (n = 13), respectively. In contrast, alignment by the first lobe greatly increased RMSD values for the non-aligned second lobe: 12.90 ± 9.10 Å for Model A and 12.87 ± 10.15 Å for Model B, reflecting much higher variability in the predicted relative positioning. It needs to be emphasized that although some flexibility of the structure would not be unexpected considering the metal-sensitive flexibility of the two lobes in GCAP1, previously shown to move around the hinge region connecting them (37, 38), the RMSD values in our modeling reflect the consistency of multiple predictions by AlphaFold rather than the actual molecular dynamics of the RetGC1:GCAP1 complex.

In approximately one-third of all Models A and B, the second semi-globule of GCAP1 was oriented toward the DD, such that the first α-helix of the second semi-globule (helix 6 in Fig. 2*A*) came in close proximity to one of the DD coils (Fig. 2*B*). In another third of the predicted structures, helix 6 and the entire second semi-globule of GCAP1 were positioned away from the RetGC1 DD domains (Fig. 2*C*). In the remaining third of the predictions, helix 6 adopted an intermediate position between the first two models. Among the three variants, only the model in which helix 6 of GCAP1 faced the DD domain was consistent with the previous biochemical evidence (27, 28) directly implicating Lys^87^ and Arg^93^ (Fig. 2, *B* and *C*) in GCAP1 as part of its binding interface for RetGC1.

Importantly, despite the variability in the position of the second semi-globule of GCAPs between the predicted RetGC1_2_:GCAP1_2_ tetramers, the overall structure of the RetGC1/GCAP1 interface remained very similar between Models A and B. According to the previous biochemical studies, multiple residues in GCAP1 creating the interface with the cyclase are predominantly located on the surface of its first semi-globule, with a few also present within the hinge region and the entering alpha-helix 6 of EF-hand 3 of the second semi-globule (Fig. 2, *A*–*C*) (28). Positions of the residues on the predicted interface between RetGC1 and GCAP1 were remarkably consistent with that biochemical data and virtually identical between the two models (Figs. 1, 2 and S2). Therefore, we arbitrarily selected, based on more ‘canonical’ structure (35) of its coiled-coil DD, Model A of the RetGC1_2_:GCAP1_2_ complex for subsequent presenting the biochemically verified structure of the interface on RetGC1.

Importantly, the overall shape of RetGC1 in the complex predicted by AlphaFold was generally consistent with the overall elongated shape of RetGC1 dimer directly observed by cryo-electron microscopy (cryo-EM) of the purified recombinant enzyme (Fig. 3). In addition, the EM map of recombinant RetGC1 fairly closely resembled the overall shape of the recently reported by Liu *et al*., 2025 (35), structure of homologous membrane guanylyl cyclase GC-A, enzyme sharing a generally similar domain architecture with RetGC1 (Fig. S3).

**Figure 3:**
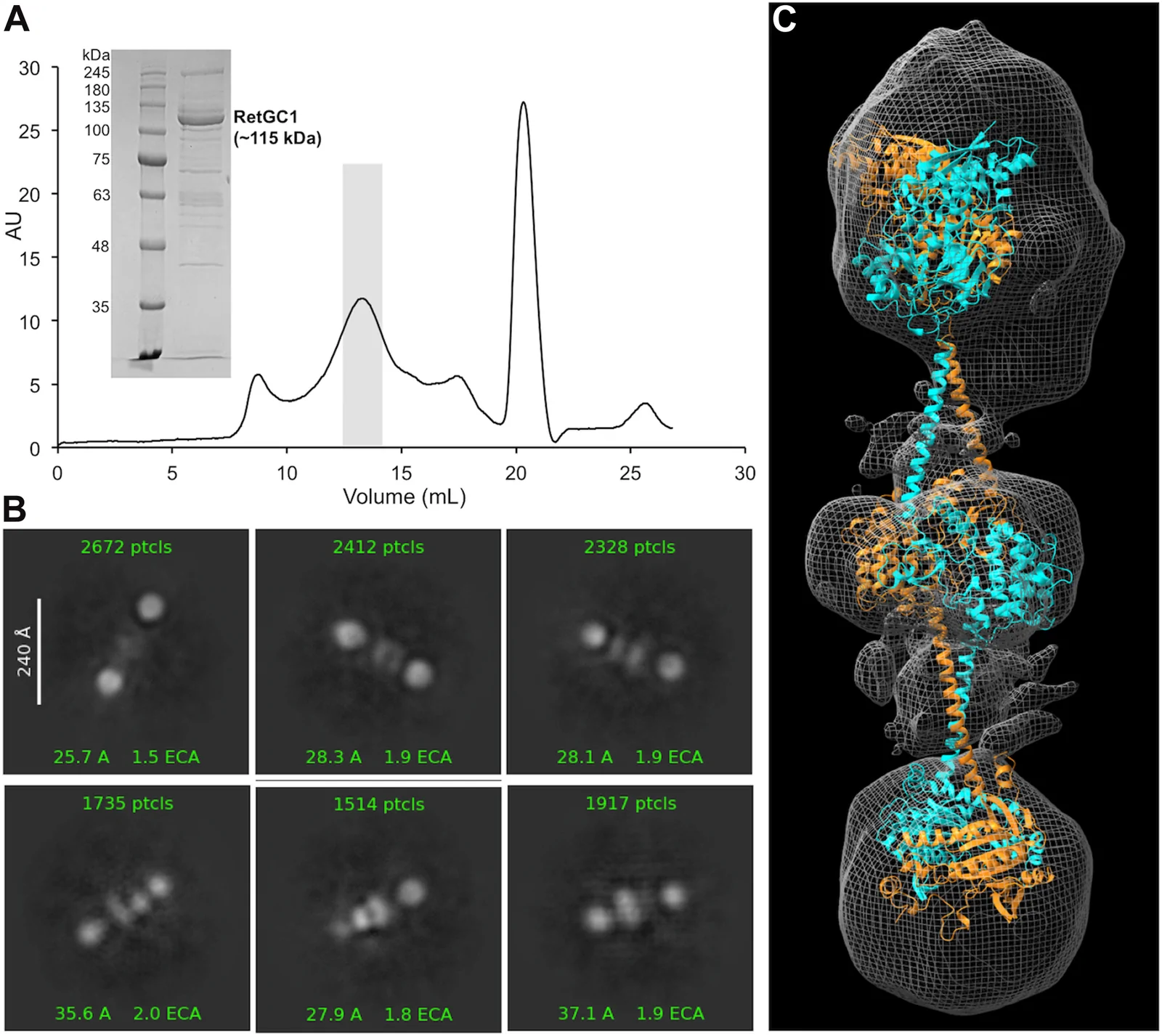
**Cryo-EM mapping of RetGC1.***A*, size-exclusion chromatography (SEC) chromatogram obtained using a Superose 6 column. The *gray-shaded* region indicates the fractions collected for cryo-EM sample preparation; *inset*–Coomassie blue-stained 9% SDS–PAGE gel showing Strep-Tactin XT 4Flow-eluted sample after the subsequent SEC step. *B*, representative 2D class averages, with the number of particles in each class indicated above the corresponding class. *C*, 11 Å cryo-EM map of the recombinant RetGC1 homodimer superimposed with the AlphaFold 3-predicted RetGC1 homodimer within the Model A of the RetGC1_2_:GCAP1_2_ tetramer.

### Site-directed mutagenesis of RetGC based on the predicted structure of the RetGC1:GCAP1 complex

On RetGC1, the predicted GCAP1-binding interface was confined to a small portion of KHD and the amino-terminal half of DD domains (Fig. 2*D* and Table 1). The residues selected for mutational analysis (shown as orange and cyan spheres in Fig. 2*D*) were all solvent exposed in both Model A and Model B and located in close proximity to GCAP1 (Table 1). Individual single-residue substitutions were introduced in RetGC1 using the strategy previously employed to identify the target-binding interfaces in GCAP1 (28) and RD3 (39). To maximize the effect of the substitutions on the interaction between GCAP1 and its target enzyme, the substituting residues altered the properties of the side chains, such as reversing the charge of the residues or changing the length of the side chain (Fig. 4 and Table 2). To assess the effect of the mutations in RetGC1 on GCAP1 binding, we evaluated apparent affinities of the mutants for GCAP1 from concentration-dependence of RetGC1 activation by GCAP1 *in vitro*. Recombinant RetGC1 was produced harboring mOrange fluorescent tag in its extracellular domain to add the capability of direct visualization of its expression in transfected HEK293 cells when required (40). This modification has already been proven not to affect regulation of RetGC1 by GCAPs (40), nor the predicted structure of RetGC1:GCAP1 interface (Fig. S4). The membrane fraction containing RetGC1 was reconstituted with purified recombinant human GCAP1 in the conditions of the assay optimal for the cyclase activation by GCAP (38), *i.e.*, in the presence of EGTA at saturating concentration of Mg^2+^ (Fig. 4). The effects of mutations in RetGC1 on its affinity for GCAP1 was evaluated based on shifting dose-dependence on GCAP1 rather than the absolute activity of the mutated cyclase, because the latter can be caused by allosterically affecting the active site of the enzyme, whereas the former would directly reflect the ability of RetGC1 to bind GCAP1.

**Figure 4:**
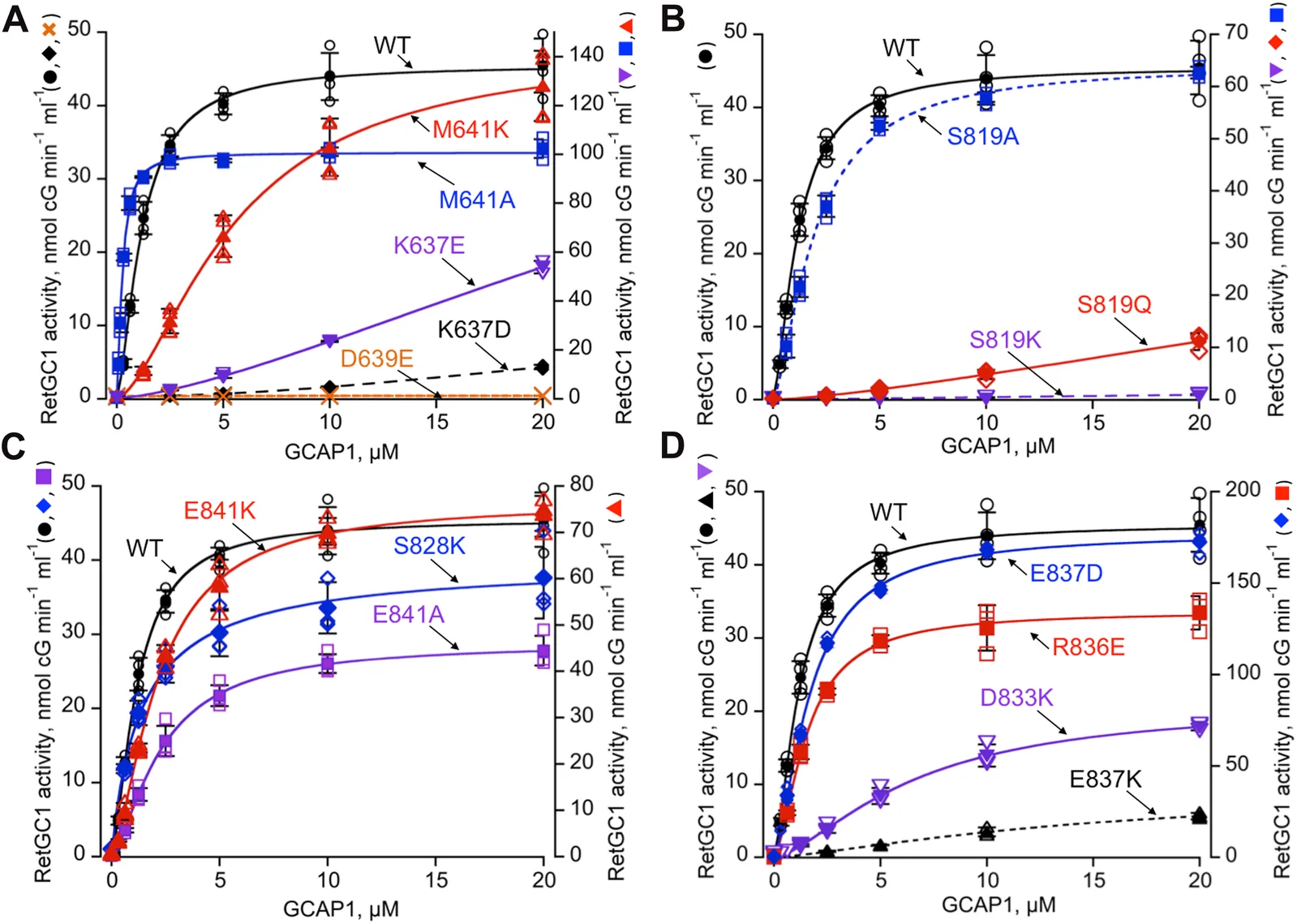
**Effect of amino acid substitutions in KHD and DD domains of RetGC1 on dose dependence of RetGC1 activation by GCAP1.***A*, mutations in KHD domain: WT (○,●); K637D (◇,◻); K637E (▽,▼); D639E (✕,✕); M641A (◻,◼); and M641K (△,▲). *B–D*, mutations in DD domain. *B*, WT (○,●); S819A (◻,◼); S819Q (◇,◻); S819K (▽,▼). *C*, WT (○,●); S828K (◇,◻); E841A (□,■); E841K (△,▲). *D*, WT (○,●); D833K (▽,▼); R836E (◻,◼); E837D (◇,◻); and E837K (△,▲). Activation of recombinant RetGC1 by human GCAP1 was assayed in the presence of 2 mM EGTA and 10 mM Mg^2+^; open symbols–individual replicates; filled symbols with error bars–mean ± SD. The data were fitted assuming a sigmoidal function, A = A_max_/(1+ ([GCAP]_half_/[GCAP])^h^), where A_max_ is the maximal activity, [GCAP] is the concentration of GCAP1 in the assay, [GCAP]_half_ is the concentration of GCAP1 producing half-maximal activation, and h is the Hill coefficient. See “Experimental procedures” for other details.

**Table 2:** The apparent affinities of RetGC1 mutants for GCAP1 in RetGC activity assay

RetGC1	*[GCAP]*_*half*_, μM (mean ± SD)^a^
Wild Type	1.18 ± 0.17 (n = 4)
K637D	ND (n = 3)
K637E	ND (n = 3)
D639E	ND (n = 3)
M641K	5.6 ± 0.17 (n = 4)
M641A	0.23 ± 0.06 (n = 4)
S819A	1.98 ± 0.1 (n = 3)
S819K	ND (n = 3)
S819Q	ND (n = 3)
S828K	1.42 ± 0.26 (n = 3)
R836E	1.57 ± 0.28 (n = 3)
D833K	6.98 ± 1.8 (n = 3)
E837D	1.68 ± 0.13 (n = 3)
E837K	15.2 ± 5.8 (n = 3)
E841A	2.3 ± 0.15 (n = 4)
E841K	2.21 ± 0.36 (n = 3)
WT + K23D GCAP1	ND (n = 3)
WT + K23E GCAP1	ND (n = 3)
E832R + WT GCAP1	4.8 ± 0.5 (n = 3)
E832R + K23D GCAP1	2.85 ± 0.77 (n = 4)
E832R + K23E GCAP1	3.81 ± 0.5 (n = 3)

a *[GCAP]_half_* is the concentration of GCAP1 producing half-maximal activation of guanylyl cyclase activity from data fit assuming the function, *A* = *A*_*max*_/(*1*+ (*[GCAP]_half_[GCAP]*)^*h*^), where *A*_*max*_ is the maximal activity, *[GCAP]* is the concentration of GCAP1 in the assay, and *h* is the Hill coefficient. See “Experimental procedures” for other details; *ND*–not determined (the apparent affinity was too low to calculate *[GCAP]_half_* from the fit).

Within the KHD domain, we examined three solvent-exposed residues predicted by AlphaFold to bind GCAP1: Lys^637^, Asp^639^, and Met^641^ (the original residue numbers in RetGC1 primary structure) (Fig. 4, Tables 1 and 2). Asp^639^ was replaced with glutamate, thereby preserving the negative charge while only minimally increasing the length of the side chain. Despite this small change, D639E RetGC1 completely lost the ability to become activated by GCAP1 (Fig. 4*A*). Cell-based assay detecting the fluorescently tagged RetGC1-GCAP1 complex in co-transfected HEK293 cells directly showed that the loss of activity was due to a dramatic reduction in the D639E RetGC1 affinity for GCAP1 (Fig. 5). The loss of D639E RetGC1 ability to form a complex with GCAP1 was specific for GCAP1, because the same mutation did not eliminate its ability to bind RD3 (Fig. 6). Similarly to the Asp^639^ substitution, a dramatic reduction of the affinity for GCAP1 resulted from substitutions of Lys^637^. Replacing the positively charged Lys^637^ with the negatively charged glutamate or aspartate markedly reduced both the apparent affinity of RetGC1 for GCAP1 in the RetGC1 activation assay (Fig. 4) and specifically disrupted co-localization of GCAP1 but not RD3 in the cell-based assay (Figs. 5 and 6).

**Figure 5:**
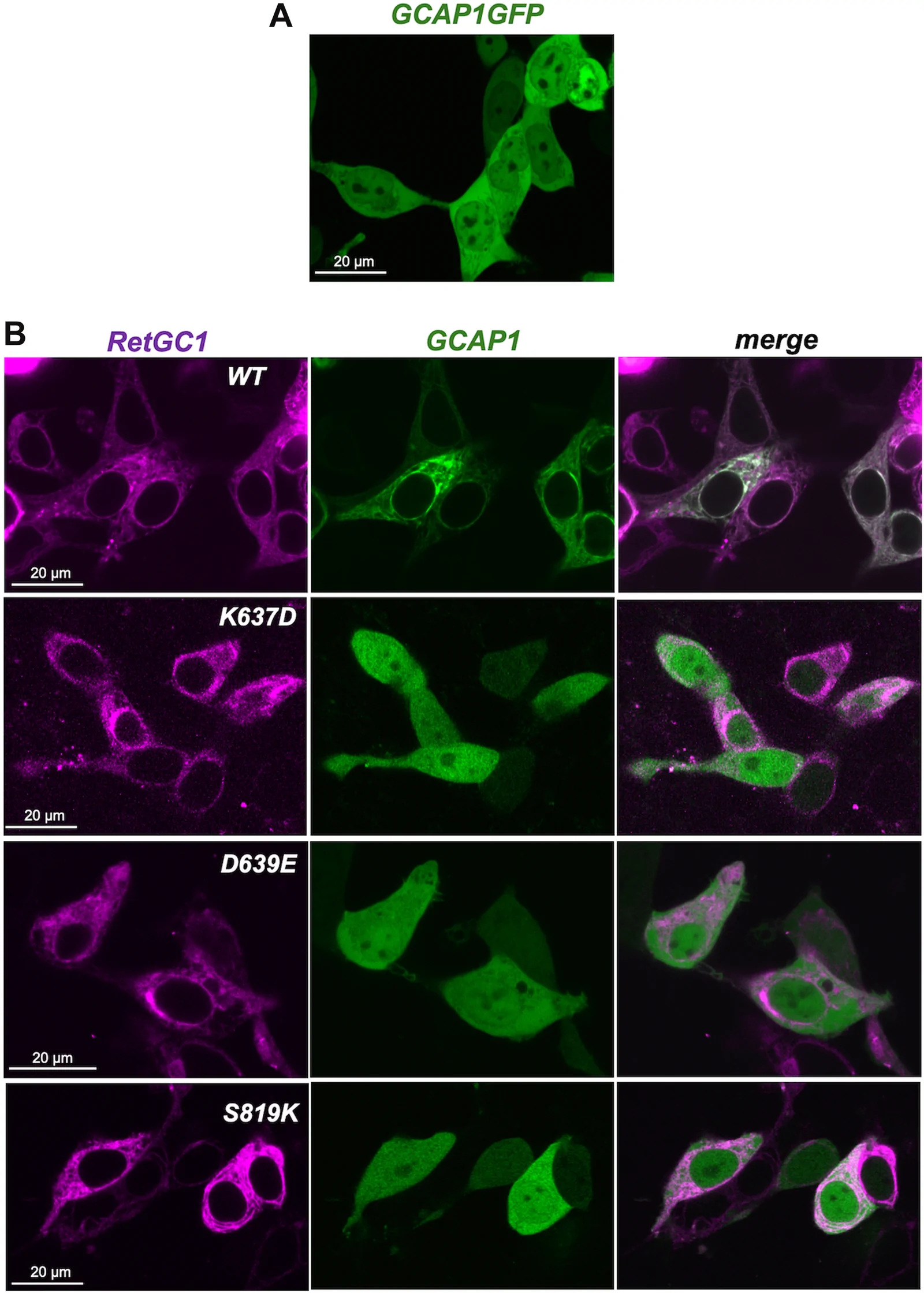
**Effect of mutations in RetGC1 on GCAP1 binding to RetGC1 in HEK293 cells.***A*, GCAP1–GFP (*green*) expressed in HEK293 cells in the absence of RetGC1 is diffusely distributed throughout the cytoplasm and the nucleus. *B*, mOrange-tagged (*magenta* pseudo-color) WT, K637D, D639E, and S819K RetGC1 co-expressed with GCAP1–GFP. Note that co-expression with WT RetGC1 results in redistribution of GCAP1–GFP, from a diffuse cytoplasmic and nuclear pattern to a void in the nuclei, cytoplasmic membrane co-localization with RetGC1; however, no GCAP1–GFP membrane co-localization is detected in the presence of K637D, D639E, or S819K RetGC1; bar–20 μm.

**Figure 6:**
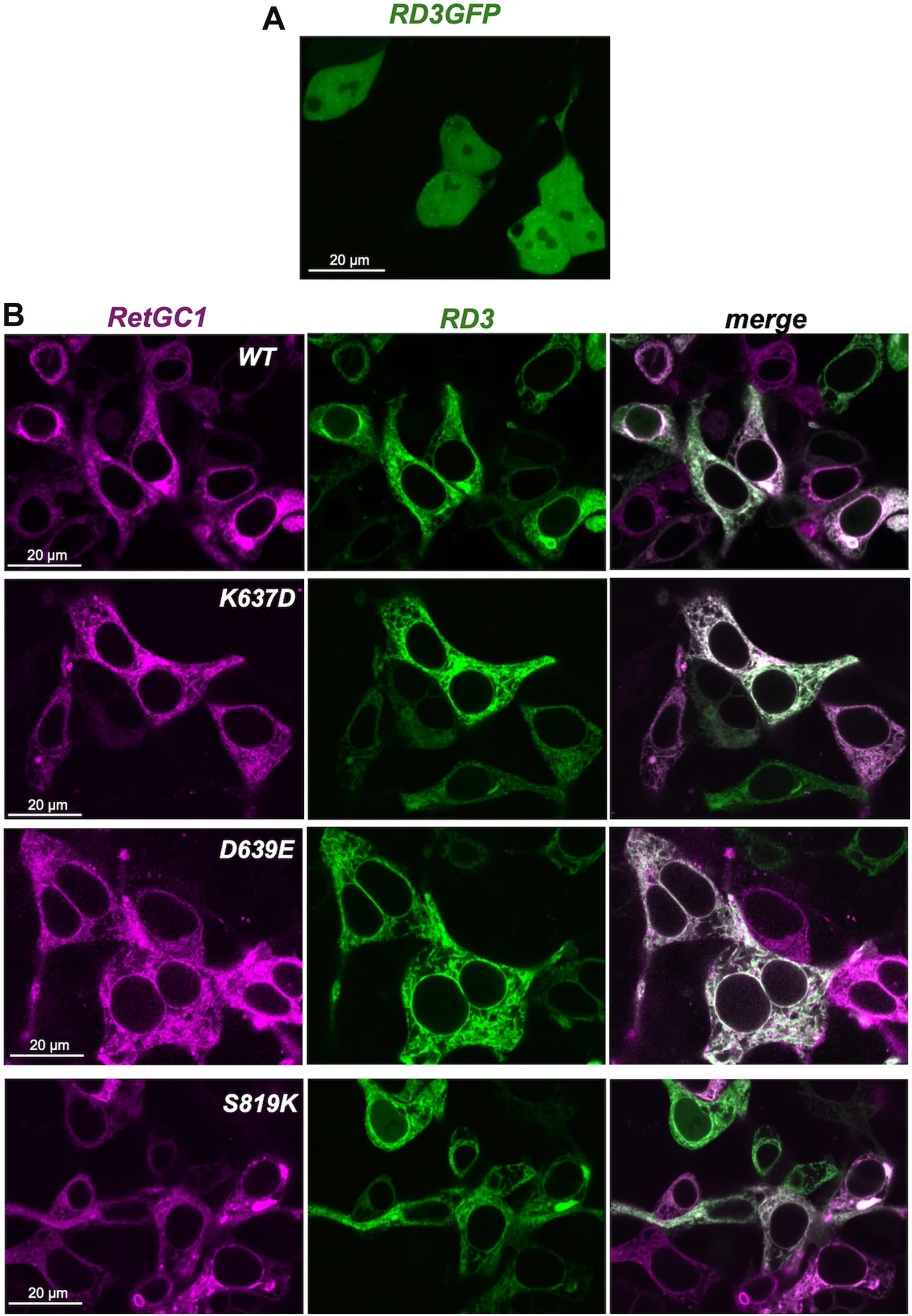
**Mutations in RetGC1 that block GCAP1 binding do not prevent binding of RD3.***A*, RD3–GFP (*green*) expressed in HEK293 cells in the absence of RetGC1 is diffusely distributed throughout the cytoplasm and the nucleus. *B*, RD3–GFP co-expression with mOrange-tagged WT, K637D, D639E, and S819K RetGC1. The rightmost panels present the merged images of the mOrangeRetGC1 fluorescence (*magenta* pseudo-color) superimposed on the green fluorescence of RD3-GFP. Note that RD3-GFP co-localizes with wild type as well as D637D, D639E, and S819K RetGC1; bar–20 μm.

To assess the importance of Met^641^, the third residue in KHD predicted to be in a close proximity to GCAP1 in the RetGC1_2_:GCAP1_2_ complex, two different substitutions were tested. Replacement with alanine, a non-polar residue with a much shorter side chain, did not disrupt binding and even increased the apparent affinity of M641A RetGC1 for GCAP1. In contrast, substitution with a polar and slightly larger side chain of lysine substantially reduced the apparent affinity of M641K RetGC1 for GCAP1, most likely by creating the expected steric hindrance (Fig. 4*A* and Table 2). This result indicates that side chain of Met^641^ *per se* is not critical for the stability of the RetGC1:GCAP1 complex but is indeed in proximity to GCAP1 close enough to make the interaction sensitive to the size of the side chain.

AlphaFold predicted that the amino-terminal portion of the DD domain also directly contacts GCAP1 (Figs. 2 and S2), consistent with previous biochemical studies (27). We therefore examined several solvent-exposed residues in this part of RetGC1 DD domain proximal to GCAP1–Ser^819^, Met^823^, Ser^828^, Glu^832^, Asp^833^, Arg^836^, Glu^837^, and Glu^841^. Three substitutions of Ser^819^ were tested for their potential effects on the apparent affinity for GCAP1. Similar to M641A, S819A replacement did not prevent binding of GCAP1, whereas both S819Q and S819K RetGC1 mutants markedly reduced their apparent affinity for GCAP1 (Fig. 4*B*) and selectively disrupted its binding of GCAP1 but not RD3 in a cell-based assay (Figs. 5 and 6). A similar effect was earlier observed in the case of Met^823^ (27), when activation of RetGC1 by GCAP1 tolerated replacement of Met^823^ with Ser, a non-charged polar residue, whereas polar residues, either negatively charged glutamate or positively charged arginine, critically compromised GCAP1 binding (27). Substitution of negatively charged Asp^833^ with lysine also weakened the apparent affinity for GCAP1 in the RetGC1 activation assay (Fig. 4*D* and Table 2), albeit to a lesser extent. Mutations in the neighboring Glu^837^ had different effect on the apparent affinity of RetGC1 for GCAP1 depending on the properties of the side chain. Preserving the charge of the side chain with only moderate decrease in size did not affect the affinity of E837D RetGC1 for GCAP1, whereas longer, positively charged lysine side chain in that position drastically reduced it (Fig. 4*D* and Table 2). For comparison, we also mutated three residues predicted to be on the periphery of the putative RetGC1/GCAP1 interface and not necessarily directly contacting GCAP1 (Fig. 3). As expected, none of the tested substitutions (S828K, R836E, E841A or E841K) substantially affected the apparent affinity of RetGC1 for GCAP1 (Table 2).

A particularly interesting effect was observed for a pair of potentially interacting side chains, Glu^832^ in RetGC1 and Lys^23^ in GCAP1. Based on the predicted structure (Fig. 3), we hypothesized that these residues could form a salt bridge (Table 1). Substitution of Glu^832^ caused a moderate reduction in the apparent affinity of E832R RetGC1 for GCAP1 (Table 2), whereas reversing the charge of Lys^23^ in GCAP1 by replacing it with either aspartate or glutamate abolished K23D and K23E GCAP1 binding to RetGC1 (Fig. 7*A*). Remarkably, combining the E83R substitution in RetGC1 with either K23D or K23E in GCAP1 effectively restored binding affinity of the mutant GCAP1 (Fig. 7*B*).

**Figure 7:**
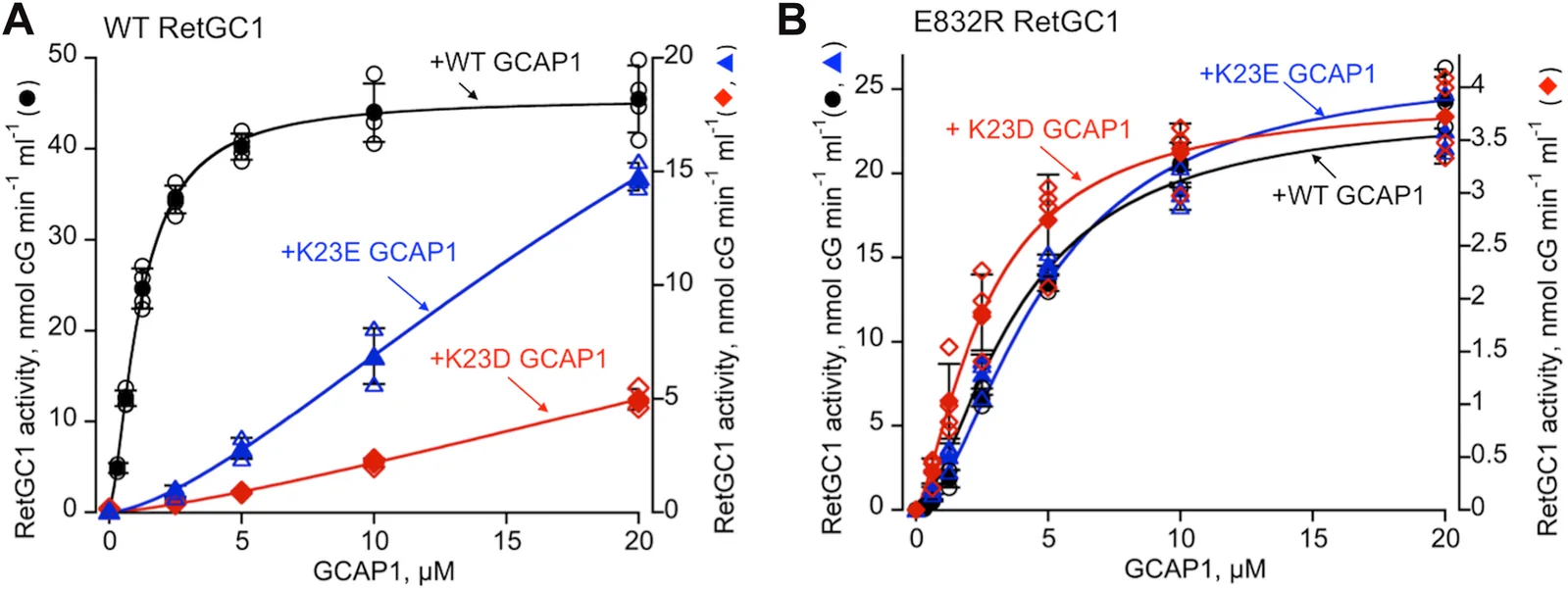
**E832R substitution in DD domain of RetGC1 restores activation of RetGC1 by K23E and K23D GCAP1.** The WT (*A*) or E832R (*B*) RetGC1 activation by WT (○,●); K23E (△,▲) or K23D (◇,◻) GCAP1 in the presence of 2 mM EGTA and 10 mM Mg^2+^; open symbols–individual replicates; filled symbols with error bars–mean ± SD; dose-dependence data fitted assuming a sigmoidal function, A = A_max_/(1+ ([GCAP]_half_/[GCAP])^h^), where A_max_ is the maximal cyclase activity, [GCAP] is the concentration of GCAPs in the assay, and h is the Hill coefficient. See “Experimental procedures” for other details.

## Discussion

Artificial intelligence–based structural predictions have emerged as powerful tools for investigating protein structure and function (31, 32). AlphaFold 3, the latest generation of the AlphaFold AI platform, utilizes deep learning–based neural network architectures trained on experimentally determined structural data to predict the three-dimensional organization of proteins and their quaternary complexes directly from amino acid sequences (32). In contrast to earlier versions optimized primarily for individual protein structures, AlphaFold 3 enables higher accuracy of modeling protein–protein interactions and multicomponent biomolecular assemblies, including conformational interaction interface regions that are difficult to resolve experimentally. In the present study, AlphaFold 3-generated structural models provided a framework for identifying the putative interaction interface between RetGC1 and GCAP1, which was subsequently validated by site-directed mutagenesis.

The RetGC1:GCAP1 complex presents a major challenge in structural studies for two main reasons. First, the RetGC1 structure was never determined by biophysical methods, such as X-ray crystallography or cryo-electron microscopy, because this dimeric membrane enzyme turns out to be a particularly difficult object for such structural analysis, even more difficult than its homolog, GC-A, whose three-dimensional structure was recently established by Liu and colleagues (35). Thus far, we were able to visualize only the overall shape of the RetGC1 dimer using a cryo-EM approach (Fig. 2). Although we continue to pursue this direction, the current cryo-EM model of RetGC1 did not provide sufficient resolution at the level of amino acid residues. Modeling of the RetGC1 catalytic domain (22) based on its homology with the catalytic domain of adenylyl cyclase was successful, and its reliability was verified in direct biochemical testing (23, 24, 33). However, biochemical evidence also argues that the catalytic domain is not directly involved in the interaction with GCAP (40). Structural model of RetGC1 was also proposed by Rehkamp and colleagues (30) based on multiple chemical cross-linking analysis, yet that model was not directly evaluated by biochemical testing of RetGC1 activity. The second reason why the structure of the RetGC1:GCAP1 complex remains unsolved by physical methods is that this complex is highly unstable in various detergents used for solubilization of RetGC1 (41), making it challenging to isolate the complex not only in its functional state but even in a state merely retaining GCAP bound to the cyclase. To date, isolation of the RetGC1:GCAP1 complex has been impossible to achieve.

Previous mutational analysis of RetGC1 strongly indicated that the most important parts of RetGC1 directly involved in its binding with GCAP1 are KHD and DD domains (27, 40). Mapping the opposite part of the interface, on the side of GCAP1, utilized global mutational analysis, in which virtually every surface-exposed residue on GCAP1 was substituted to probe for its involvement in binding RetGC1 (28). Importantly, that approach relied on the already determined by Stephen and colleagues (36) X-ray crystallography structure of GCAP1. Such global mutagenesis would not be feasible in the case of a much larger RetGC1 molecule in the absence of any actual structural information. In that case, loss of GCAP1 binding resulting from random substitutions in RetGC1 could merely reflect non-specific misfolding of RetGC1 rather than point at the residues that create the interface. Hence, instead of using a ‘blindfolded’ random mutational analysis in order to map the interface on RetGC1 for GCAP1, we chose to utilize AphaFold-guided amino acid substitutions in RetGC1. It is important to emphasize that we used this approach to identify the residues in RetGC1 and GCAP1 forming their binding interface, rather than to claim that we esatblished a complete detailed structure of the complex.

There are several reasons to consider the AlphaFold-generated structure of RetGC1_2_:GCAP1_2_ tetramer to be relevant to that purpose. The AlphaFold 3 algorithm only utilizes the primary structures of RetGC1 and GCAP1 for calculating its quaternary structure (31, 32). The preferred relative positions of GCAP and RetGC1 subunits are not among the input parameters used for calculating the structure. Nonetheless, despite minor differences between the outcomes of the individual models, the resultant structures in multiple independent trials of AlphaFold predictions consistently position GCAP1 in contact with the KHD and DD domains of RetGC1, domains that were independently predicted in biochemical experiments (27, 40) to be the most likely parts of RetGC directly involved in GCAPs binding. Secondly, GCAP1 in these models consistently made contacts with RetGC1 *via* residues predominantly located in its first semi-globule, including EF-hand 1 and EF-hand 2, implicated in previous biochemical studies in forming the interface with RetGC1 (28). Last but not least, out of curiosity, we used AlphaFold 3 to predict the 3D structure of a peptide hormone receptor guanylyl cyclase GC-A a few months before the structure of GC-A was established. The structure predicted by AlphFold 3 was nearly identical to the actual cryo-EM structure more recently reported by Liu and colleagues (35) (Fig. S3*B*). Both enzymes have a similar organization of the intracellular domains except for the position of KHD domain relative to the DD domain (Fig. S3, *B* and *C*). Yet, including GCAP1 primary structure in the input for the modeling potential quaternary structure completely failed to generate a complex containing GC-A and GCAP, which was also consistent with earlier biochemical findings that GC-A, in contrast to RetGC1, does not bind GCAP1 (40).

Site-directed mutagenesis of RetGC1 guided by AlphaFold confirms several key residues in KHD and DD domains as parts of the interface with GCAP1. Identification of the residues making the contact with GCAP1 (Figure 4, Figure 5, Figure 6, Figure 7 and Table 2) validates the general AlphaFold model (Figs. 1, 2 and 8). Asp^639^ in KHD was predicted to play one of the most critical roles in GCAP1 binding by interacting with Cys, Pro, Ser, and Gly in the loop of the non-metal binding EF-hand 1 of GCAP1 (Fig. 2). Indeed, even minimal elongation of the side chain by a single CH_2_ group completely disrupts binding of GCAP1, but not binding of RD3 (Figure 4, Figure 5, Figure 6), indicating that Asp^639^ is specifically a part of the RetGC1 interface with GCAP1. Interestingly, a substitution of Asp^639^ suppressing RetGC1 activation was also found in LCA patient (26). In addition to Asp^639^ in KHD domain, Ser^819^ in DD domain predicted to be a part of the interface (Fig. 2 and Table 1) has been confirmed as such by biochemical testing (Figs. 4, 5 and Table 2).

We also find as the most intriguing outcome of the prediction and verification of the RetGC1/GCAP1 interface in the AlphaFold model that each GCAP1 molecule in RetGC1_2_:GCAP1_2_ tetramer binds to KHD of one RetGC1 subunit and to DD domains on both subunits (Fig. 8). Earlier observations that GCAPs can form dimers *in vitro* (42, 43, 44) lead to a hypothesis, that association and dissociation of GCAP dimer may control the dimerization state of the cyclase. The AlphaFold-generated model biochemically verified in the present study indicates that GCAP1 does not need to form the GCAP1:GCAP1 dimer in order to regulate the cyclase. Since each GCAP molecule, independently of each other, binds to both subunits of RetGC1 homodimer, conformational changes in a monomeric GCAP1 caused by binding and release of metal ions should be able to directly affect the coiled-coil of DD domain. This possibility agrees with the earlier evidence that binding of a single Mg^2+^-liganded GCAP1 molecule to RetGC1 dimer can increase the activity of the cyclase, even though not as effectively as 2 Mg^2+^GCAP molecules (27).

**Figure 8:**
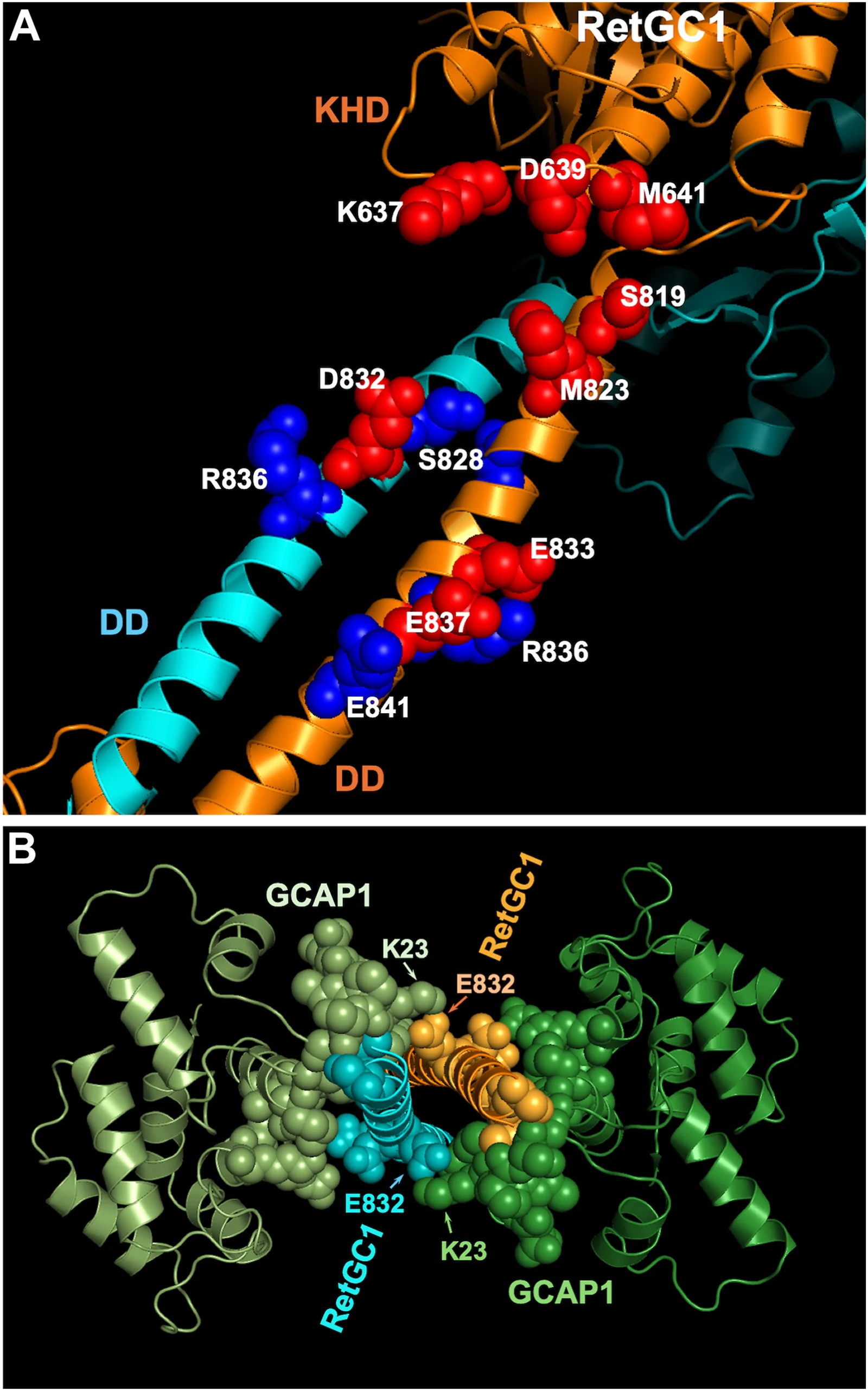
**Interface with GCAP1 on RetGC1 suggests regulation of the RetGC1 activity by affecting structure of the cyclase dimer *via* DD domain.***A*, residues in RetGC1 biochemically confirmed to participate in GCAP1 binding are shown in *red*, whereas residues that are on the periphery of the interface and do not critically affect GCAP1 binding are shown in *blue*, the DD domains in two subunits of the RetGC1 dimer are marked in *cyan* and *orange*. *B*, in RetGC1_2_:GCAP1_2_ tetrameric complex, each of the two GCAP1 molecules (*light green* and *dark green*) binds DD domains in both RetGC1 subunits (only DD domains of RetGC1 subunits are shown in the diagram for clarity). See Discussion for other details.

Importantly, the manner in which GCAP1 binds to both subunits of RetGC1 homodimer explains why even relatively subtle changes in the conformation of GCAP between its RetGC1-inhibiting, Ca^2+^-liganded, and RetGC1-activating, Mg^2+^-liganded, states (45, 46) can cause a dramatic change in the rate of GTP to cGMP conversion in the active site of the enzyme (Fig. 8), without the need for reversible dimerization of GCAP1 itself. It has been shown that binding of natriuretic peptide hormone to the extracellular domain of GC-A (35) translates, through the changes in the intracellular portion of the molecule, to changing the conformation of the dimeric catalytic domains forming the active site of the enzyme. We propose that, in a manner similar to the change in catalytic domain in GC-A, the conformational changes in each GCAP1 molecule upon exchange of Mg^2+^ for Ca^2+^ and *vice versa* in EF-hands 3 and 4 (1, 38) directly exert pull-push actions on the opposite subunits of RetGC1. While each GCAP1 is anchored to Asp^639^ and Ser^819^ on one RetGC1 subunit, Lys^23^ in its EF-hand 1, *via* contact with Glu^832^ in the DD RetGC1 domain of the opposite subunit, could convert Ca^2+^/Mg^2+^-dependent conformational changes in GCAP1 into conformational changes of the coiled-coil DD domain and consequently affect the dimerized catalytic domain of RetGC1 (22) downstream of the DD, thus activating or deactivating GTP to cGMP conversion in the active site.

It needs to be noted, however, that AlphaFold 3 modeling also has certain limitations that do not allow for comparison between the activated and the inhibited states of the predicted RetGC1_2_:GCAP1_2_ complex. AlphaFold algorithm for predicting complex protein structures (32) is practically insensitive to single amino acid substitutions introduced in protein primary structure (47). Therefore, the structural changes resulting from the point mutations tested in this study could not be directly evaluated. Our attempts to include Mg^2+^ and Ca^2+^ in the input for modeling also did not produce specific changes in the predicted structure of the tetramer and often randomly positioned metal in various parts of the structure. Uncertainty also remains with the orientation of the second semi-globule of GCAP1 in the complex, which may be critically important for the regulation of the cyclase between the Mg^2+^ and Ca^2+^-liganded states of GCAP1 (38). Perhaps future development of the AI-based structural predictions by AlphaFold would be able to resolve the subtle conformational changes underlying the regulation of RetGC1 activity by GCAP1. However, we reason that the structure of the complex predicted by AlphaFold 3 fairly adequately reflects the overall structure of the RetGC1_2_:GCAP1_2_ tetramer. Direct comparison with the structure obtained by physical methods, when becomes available, would help further evaluate the prediction accuracy of AlphaFold.

## Experimental procedures

### AlphaFold 3 modeling

Structural models of the RetGC1–GCAP1 complex were generated using AlphaFold 3 with default settings ((32); https://alphafoldserver.com/). Amino acid sequences of the full-length human RetGC1, including the leader peptide and the human GCAP1 were used as the input.

### Cryo-electron microscopy (cryo-EM)

RetGC1 containing a Strep tag downstream of the signal cleavage site was expressed in HEK293F cells transfected with 1 μg of pCDNA3.1-StrepGC for each 1 × 10^6^ cells with polyethylenimine (PEI) in a 3:1 ratio. Cells (∼10 g) were resuspended in resuspension buffer (25 mM HEPES, pH 7.4, 150 mM NaCl, and 1 mM dithiothreitol (DTT)) containing benzonase (Sigma-Aldrich) and protease inhibitor (1:1000) (Millipore) and solubilized in 1% n-dodecyl β-D-maltoside (DDM) and 0.2 cholesteryl hemisuccinate (CHS) at 4 °C for 2 h. After centrifugation at 142,000*g* for 60 min in a Beckman Ti45 rotor, the supernatant was applied to a Strep-TactinXT matrix, washed with 15 column volumes of wash buffer (25 mM HEPES, pH 7.4, 150 mM NaCl, 1 mM DTT, 0.1% DDM, 0.02% CHS) and subsequently washed with SEC buffer (25 mM HEPES, pH 7.4, 150 mM NaCl, 1 mM DTT, 0.04% glyco-diosgenin (GDN)) to exchange DDM with GDN. Bound protein was eluted using 50 mM Biotin in SEC buffer at 4 °C. The eluent was concentrated in 100 kDa cutoff (Millipore) concentrator and further subjected to SEC on a Superose 6 column (GE Healthcare) in the SEC buffer. The fraction containing RetGC1 was concentrated and mixed with 1 mM ATP and 2 mM MgCl_2_. The sample was applied to a glow-discharged 300-mesh R1.2/1.3 UltrAuFoil and plunge-frozen using a Vitrobot Mark IV (Thermo Fisher Scientific, FEI). Data collection was done on a Titan Krios electron microscope equipped with a Falcon 4i detector operating at 300 kV. EER files were collected at a nominal magnification of 130,000× in electron-counting mode at 0.96 Å/pixel. Defocus ranged from −0.5 to −3 μm with a total dose of 50 e^−^/Å^2^. A single grid with 6200 movies was collected.

Data were processed using Cryosparc 4.5.1 (48). The movies were corrected using patch motion correction and patch CTF fitting. Particles were picked *via* template picking with a particle diameter of 512 Å using RetGC1 AlphaFold2 model as template. Particles were extracted and 2D classification was performed prior to multiple rounds of *ab initio* and 3D heterogenous refinement in C1 symmetry to identify RetGC1-like particles. Maps generated with nonuniform refinement were used for analysis in ChimeraX (49).

### DNA constructs and mutagenesis

The mOrange-tagged human RetGC1 construct, was expressed from a modified pRCCMV vector under the control of the cytomegalovirus (CMV) promoter, as described previously (27, 40). In this construct, mOrange cDNA replaced a segment of the extracellular domain of RetGC1, which does not affect RetGC1 activity or GCAP-dependent regulation (40). GCAP1 variants used for biochemical assays were expressed from a pET11d vector (originated from Novagen/Calbiochem). GCAP1-GFP and RD3-GFP used for cell-based co-localization assays were expressed from a pQBIfN3 vector (Clontech), in which GCAP1 or RD3 is tagged at the C-terminus with SuperGlo enhanced GFP, as described previously (28, 40, 50). The indicated substitutions were introduced into RetGC1 and GCAP1 cDNAs by replacing the corresponding wild-type cDNA fragments with synthetic double-stranded DNA fragments harboring the desired mutations (produced by Integrated DNA Technologies or Azenta). The final expression constructs were verified by Sanger sequencing.

### Recombinant RetGC1 expression and activity assay

The mOrange-tagged wild-type and mutant RetGC1 were transiently expressed in HEK293 cells (GIBCO/Invitrogen) from a modified pRCCMV vector using calcium-phosphate DNA precipitation method described previously (51). Cells were transfected with approximately 20 μg of plasmid DNA per 100-mm culture dish, incubated in 5% CO_2_ at 37 °C, and harvested 24 h after transfection. Cells were detached from the dishes, resuspended in cold phosphate-buffered saline and pelleted by centrifugation at 2000*g* for 10 min, 4 °C. The cells were resuspended in cold hypotonic buffer A containing 10 mM Tris-HCl, pH 7.6, 2 mM MgCl_2_, 1 mM DTT, 0.5 mM ATP, and protease inhibitor cocktail (Millipore/Sigma), lysed on ice in a Dounce homogenizer (Pestle A), and subjected to differential centrifugation, first at 900*g* for 5 min, 4 °C, to remove nuclei and large aggregates, then membrane fraction was collected at 23,000*g* for 20 min, 4 °C. The membrane pellet was resuspended in buffer A containing 10% glycerol, aliquoted, frozen in liquid nitrogen, and stored at −70 °C. The content of RetGC1 in different membrane preparations was equalized by SDS-PAGE and immunoblotting using a rabbit polyclonal antibody (52) against Met^797^-Ser^1103^ fragment of human RetGC1. The activity of the cyclase was assayed using [α–^32^P]GTP as a substrate (Revvity) and the [^32^P]cGMP product was quantified as described previously (53). Briefly, the assay mixture (25 μl) containing HEK293 membranes in 30 mM MOPS–KOH (pH 7.2), 60 mM KCl, 4 mM NaCl, 1mM DTT, 2 mM EGTA, 10 mM MgCl_2_, 0.3 mM ATP, 4 mM cGMP, 1 mM GTP, ∼1 μCi of [α–^32^P]GTP, 100 μM zaprinast and dipyridamole, 5 mM creatine phosphate and 0.5 unit of creatine phosphokinase (Sigma-Aldrich/Millipore) was incubated at 30 °C for 40 min and the reaction was stopped by heat-inactivation at 95 °C for 2.5 min. The [^32^P]cGMP product separated by TLC on fluorescently backed polyethyleneimine cellulose plates (Merck) in 0.2 M LiCl was eluted in 2M LiCl and counted using liquid scintillation. Data fitting and analysis was performed using a Synergy Kaleidagraph 4 software.

### Human GCAP1 expression and purification

Myristoylated human GCAP1 and its mutant variants were expressed from the pET11d (Novagen/Calbiochem) vector in *E. coli* BLR(DE3) strain harboring the pBB131 plasmid encoding yeast N-myristoyltransferase (54). The proteins were then extracted from inclusion bodies using 8 M urea, refolded by dialysis, and purified by butyl-Sepharose and size-exclusion chromatography as previously described in detail (52), except that the step converting the purified GCAP1 to the Ca^2+^-free state was omitted.

### Cell-based assay of RetGC1 binding to GCAP1 and RD3

Co-localization of GCAP-GFP and RD3-GFP with mOrangeRetGC1 variants was tested using a previously described HEK293 cell-based assay as described originally (55) with modifications described in (27, 40, 53). HEK293 cells grown in Lab-Tek chambered coverglass slides in 5% CO_2_ at 37 °C were co-transfected using a Promega FuGENE HD transfection reagent with a mixture of mOrangeRetGC1 coding plasmid and 1/200 M ratio of a plasmid harboring either GCAP-GFP or RD3-GFP cDNA. Confocal images were taken 24 to 48 h after the transfection using an Olympus FV1000 Spectral instrument. The red and green fluorochromes were excited sequentially at 543 and 488 nm, respectively, and images were processed using an Olympus FluoView FV10-ASW software. Minor contrast and gamma correction, were applied only to whole images to enhance image presentation in print.

### Protein model

The RetGC1_2_:GCAP1_2_ tetramer model generated by AlphaFold 3 was presented and analyzed using a Shröedinger PyMol 3.1.4.1 software. The model of the complex has been deposited to ModelArchive (Deposition to Computational Macromolecular Structural Models, https://modelarchive.org/) (56); https://modelarchive.org/doi/10.5452/ma-vvbdm.

### Data availability

The data referred to in this manuscript are contained within the manuscript and the Supplement. Unprocessed data can be available upon reasonable request from the corresponding author (alexander.dizhoor@drexel.edu).

## Supporting information

This article contains supporting information.

## Conflict of interest

The authors declare that they have no conflicts of interest with the contents of this article.
