# Supplementary material for: The kinase homology and dimerization domains in retinal membrane guanylyl cyclase 1 form the binding interface for the calcium sensor GCAP1
Source: J Biol Chem. 2026 Sep 25;302(11):113606. doi: 10.1016/j.jbc.2026.113606 (PMC13720810; doi:10.1016/j.jbc.2026.113606)
Supplement: Supplementary file 1 [file mmc1.pdf]

SUPPLEMENT

The kinase homology and dimerization domains in retinal membrane guanylyl cyclase 1 form the binding interface for the calcium sensor GCAP1

Igor V. Peshenko, Lyuqi Tan, Jai Mahto, Filip Van Petegem, Robert S. Molday, and Alexander M. Dizhoor

Journal of Biological Chemistry, 2026, 113606; https://doi.org/10.1016/j.jbc.2026.113606

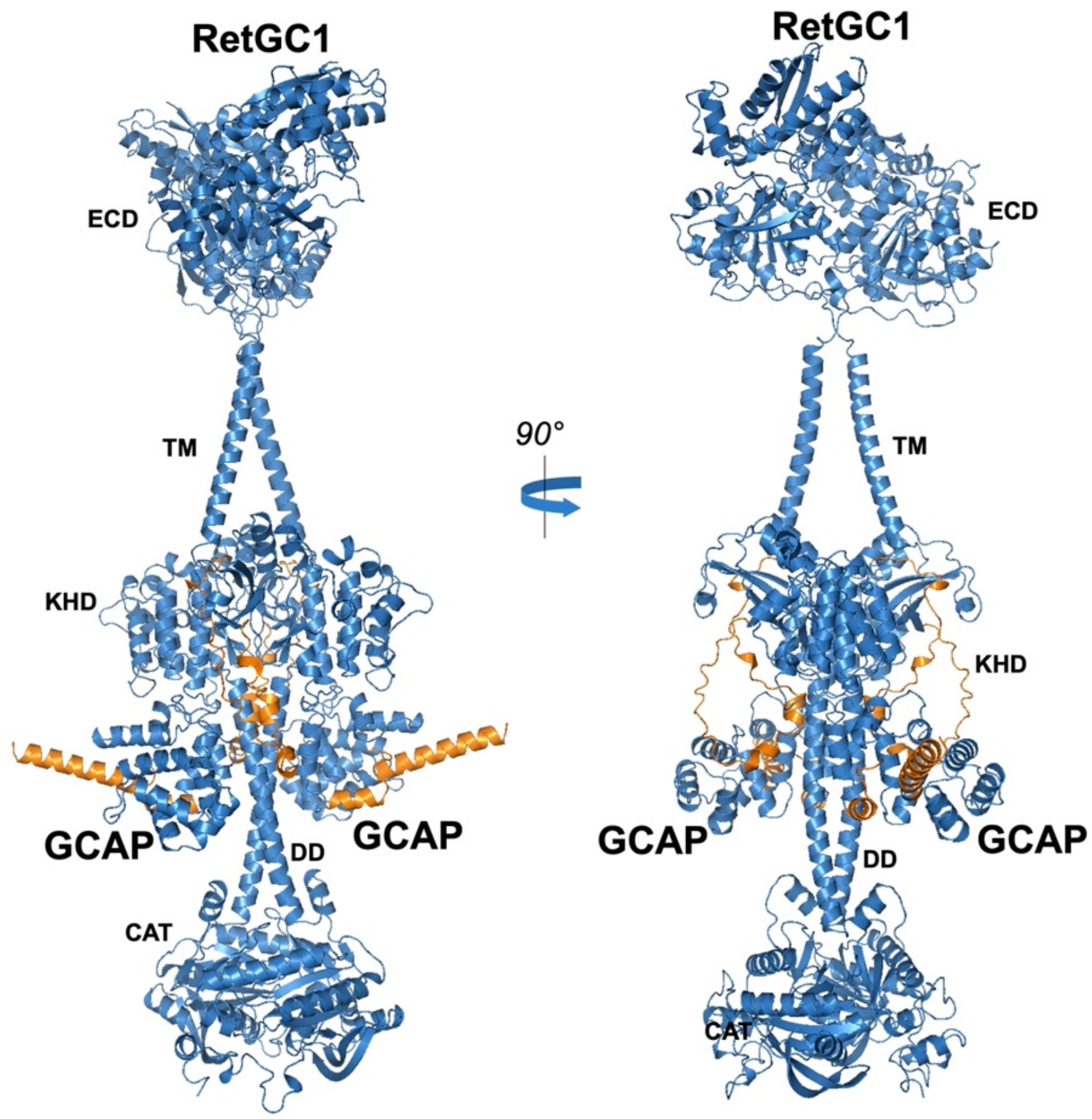

Supplemental Figure S1. **Low-confidence regions in AlphaFold-predicted structure of RetGC1$_2$:GCAP1$_2$ complex.** Regions in RetGC1 and GCAP1 structures downstream from RetGC1 TM domain colored in *blue* were consistently predicted with higher confidence (pLDDT score > 70; reference 32). The cytosolic portion of RetGC1 and GCAP1 regions with pLDDT score > 70 were included in modeling and biochemical verification of the interface; those regions where the structures were consistently predicted with lower confidence (pLDDT score < 70, colored in *orange*) were not included in model analyses. *ECD*, 'extracellular' (intradiscal) domain; *TM*, transmembrane domain; *KHD*, kinase homology domain; *DD*, dimerization domain; *CAT*, catalytic domain

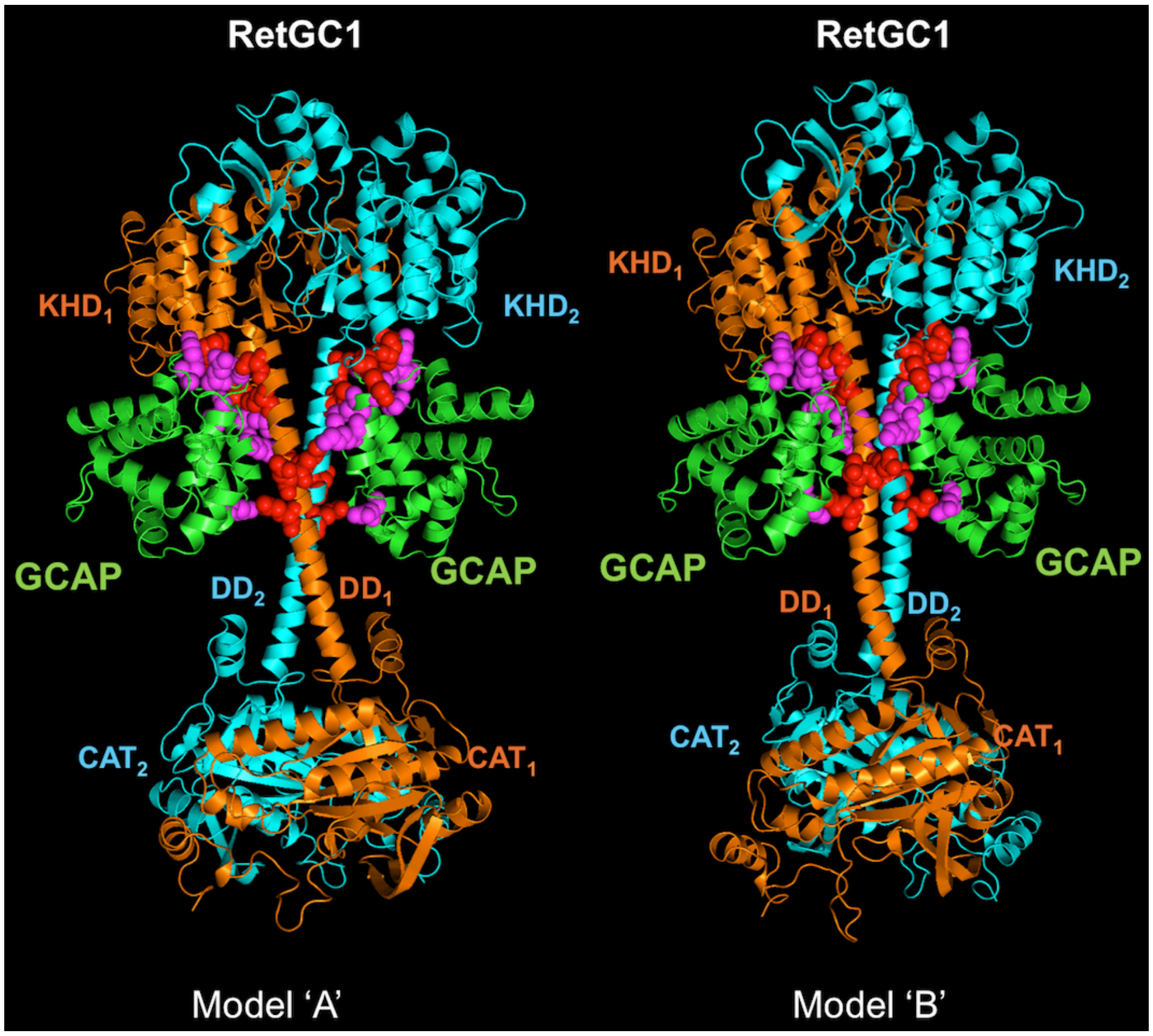

Supplemental Figure S2. GCAP1:RetGC1 interface in the cytoplasmic portions of the representative Model 'A' (*left*) and Model 'B' *(right*) of the RetGC1$_2$:GCAP1$_2$ complex generated by AlfaFold 3 depicting GCAP1 molecules (*green*) bound to the cytoplasmic portion of the RetGC1 homodimer containing KHD, DD, and CAT domains. The shape of the coiled-coil DD domain in RetGC1 is different between the two models, but the same residues in KHD and DD domains, Lys^{637}, Asp^{639}, Met^{641}, Ser^{819}, Met^{823}, Glu^{832}, Asp^{837} (*red spheres*) are in close proximity to GCAP1 residues Lys^{23}, Met^{26}, Glu^{28}, Ser^{31}, Gln^{33}, Tyr^{70}, Gln^{90} (*magenta spheres*) in both models.

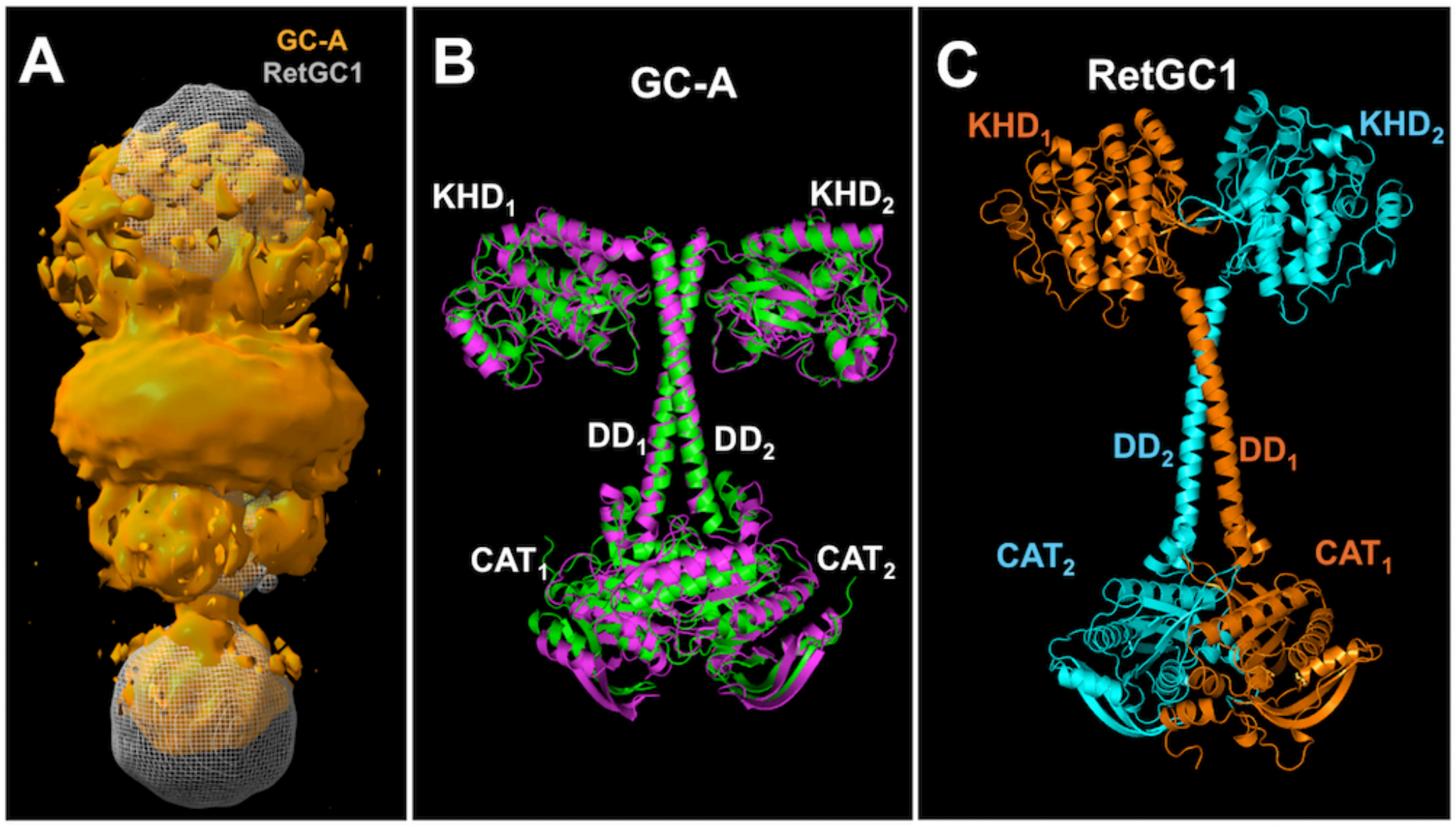

Supplemental Figure S3. ***A,*** resolution-matched comparison of GC-A and RetGC1 cryoEM maps. The GC-A EM (35) map (*orange*) was low-pass filtered to 12 Å using Volume Tools in CryoSPARC and superposed onto the RetGC1 EM map (*gray*), revealing their similar overall shape. ***B***, the AlphaFold-predicted structure of the cytoplasmic segment of GC-A (green) containing KHD, DD, and CAT domains superposed in PyMOL to experimentally determined cryo-EM structure of the GC-A dimer (PDB: 9BCO (35); *magenta*), aligned to KHD domains. The AlphaFold 3 prediction was generated before the actual cryo-EM structure (35) was released. Full-length GC-A protein sequence was used as the input for AlphaFold modeling. ***C***, representative AlphaFold-generated structure of the cytoplasmic portion of RetGC1 from the RetGC1:GCAP1 complex (‘Model A’); the two RetGC1 subunits are shown in *orange* and *cyan*; GCAP1 molecules are omitted.

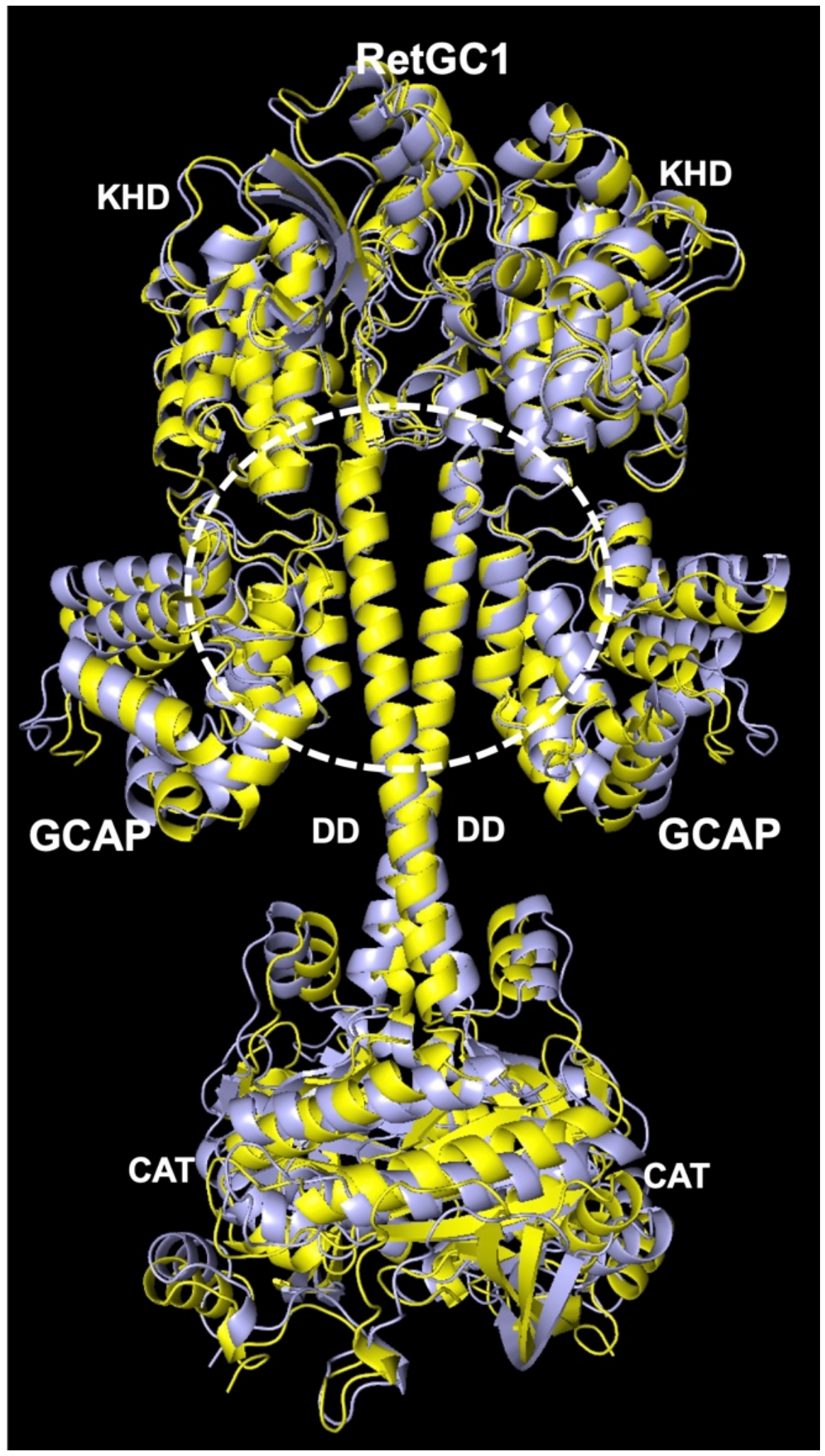

Supplemental Figure S4. **Extracellular tag on RetGC1 does not change position of the AlphaFold-predicted RetGC1/GCAP1 interface.** The cytoplasmic segment of the untagged RetGC1 in complex with GCAP1 ('Model A', *gray*) superimposed on mOrangeRetGC1$_2$:GCAP1$_2$ tetramer (*yellow*); dashed oval–the region containing predicted RetGC1/GCAP1 interface.
